# Multifunctional Rhizobacterial Consortia Enhance Growth and Yield of Common Bean Across Field Conditions

**DOI:** 10.1111/1758-2229.70414

**Published:** 2026-09-17

**Authors:** Ana Paula Santos Oliveira, Cássia Cristina Rezende Mirza, Cleiton Mateus Sousa, Enderson Petrônio de Brito Ferreira

**Affiliations:** ^1^ Universidade Federal de Goiás Goiânia Goiás Brazil; ^2^ Instituto Federal Goiano – Campus Ceres Ceres Goiás Brazil; ^3^ Embrapa Arroz e Feijão Santo Antônio de Goiás Goiás Brazil

**Keywords:** bioinputs, grain yield, inoculants, *Phaseolus vulgaris* L.

## Abstract

The common bean holds significant economic and social importance, with Brazil being one of the largest producers and consumers worldwide. Its cultivation requires high soil fertility, leading to the intensive use of mineral fertilizers, with high costs and environmental impacts. An alternative is the application of plant growth‐promoting rhizobacteria, which have the potential to ensure grain yield. The aim of this study was to evaluate the agronomic performance of common beans in response to consortia of multifunctional rhizobacteria. The experiments were conducted using a randomized block design with 22 treatments and four replications. Each treatment consisted of a combination of three PGPR strains, along with zero fertilizer and fertilizer‐only control treatment. Evaluations were carried out at the beginning of flowering and at harvest to assess root system characteristics, shoot development, and yield components. The consortia *Rhizobium* sp. (PaJuG2R5) + *Bacillus pumilus* (BRM063573) + *Stenotrophomonas maltophilia* (BRM063574), *Rhizobium* sp. (PaJuG2R5) + *Bacillus subtilis* (BRM067207) + *Bacillus pumilus* (BRM067206), and *Rhizobium* sp. (TilvG1R2) + *Bacillus pumilus* (BRM063573) + *Bacillus pumilus* (BRM067206) promoted plant growth and development, resulting in increased grain yield. These results offer a sustainable and accessible strategy for family farming, reducing dependence on mineral fertilizers, production costs, and environmental impacts.

## Graphical abstract

Consortia of plant growth‐promoting rhizobacteria (PGPR) enhance the growth and development of common bean plants, improving root system development, nutrient use efficiency and grain yield. These bioinputs reduce dependence on mineral fertilizers and support eco‐efficient agriculture, offering a sustainable alternative in diverse soil and climate conditions.

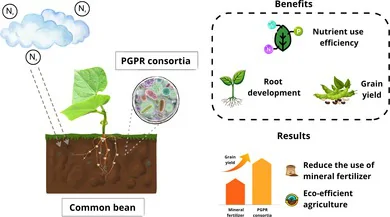

## 1 Introduction

In recent years, sustainable technologies have made significant progress in agriculture worldwide. However, their adoption in common bean cultivation remains limited, particularly in regions where production is predominantly carried out by family farming (Ganascini et al. 2019). Despite Brazil's high bean production, especially in the Cerrado region, yields are often constrained by nutrient scarcity and the limited adoption of modern technologies, resulting in an average grain yield of 1138 kg ha^−1^ (Conab 2024).

To reduce reliance on mineral fertilizers in common bean production and ensure both economic and environmental benefits, several bacterial species have been investigated. Many of these microorganisms are classified as plant growth‐promoting rhizobacteria (PGPR), which enhance plant development through direct and indirect mechanisms, including biological nitrogen fixation, phosphate solubilization and phytohormone production. These functional traits contribute to improved nutrient acquisition, plant vigour and grain yield (Filipini et al. 2021).

Based on the multifunctionality of PGPR, the development of bacterial consortia has emerged as a key strategy to enhance the sustainability of bean production systems and to improve grain yield. This approach can potentially overcome the limitations associated with single‐strain inoculants, offering greater stability under adverse environmental conditions (Ram et al. 2022).

The main challenge lies in establishing synergistic interactions among bacterial strains with diverse mechanisms of action, nutritional requirements and growth characteristics, while also considering plant‐specific needs and variable soil and climatic conditions. These consortia aim not only to reduce application costs but also to increase biodiversity in the rhizosphere, thereby fostering a more competitive, economically viable and sustainable agricultural system (Andrade et al. 2023). In this context, the multifunctional PGPR consortia combining complementary mechanisms of action would promote common bean growth and yield more consistently than mineral fertilization or single‐strain inoculants, particularly under variable field conditions.

Accordingly, the aim of this study was to evaluate the agronomic performance of common beans in response to multifunctional rhizobacteria under field conditions.

## 2 Material and Methods

### 2.1 Characterization of the Experimental Areas

The experiments were conducted in different locations over three agricultural seasons. One experiment took place in the municipality of Ceres, GO (15°21′16″ S, 49°36′23″ W, 560 m altitude) during the 2022 winter season. The others were carried out in the municipality of Santo Antônio de Goiás, GO (16°29′16″ S, 49°17′56″ W, 772 m altitude) during the 2022 and 2023 winter seasons, as well as the 2022/2023 rainy season. The soil in the Ceres area is classified as an Eutrophic Red Latosol, while the soil in Santo Antônio is classified as a Typical Acric Red Latosol, both with a clayey texture.

The land use history in the Ceres area includes successive corn crops and a first soybean crop under conventional tillage. Beans were subsequently grown after soybeans, following a four‐month fallow period without soil disturbance. In Santo Antônio, the experiments were carried out in different quadrants of the same pivot area. The land use history there includes soybean/bean cultivation in the first season, cover crops with *Brachiaria ruziziensis* in the second season, and beans in the third season.

The rainfall pattern in both locations is well defined, with a rainy season from October to April and a dry season from May to September. Average annual rainfall ranges from 1460 to 1700 mm. According to the Köppen climate classification, the regions have a tropical savanna climate (Aw), characterized by a dry winter (Köppen and Geiger 1930).

**FIGURE 1:**
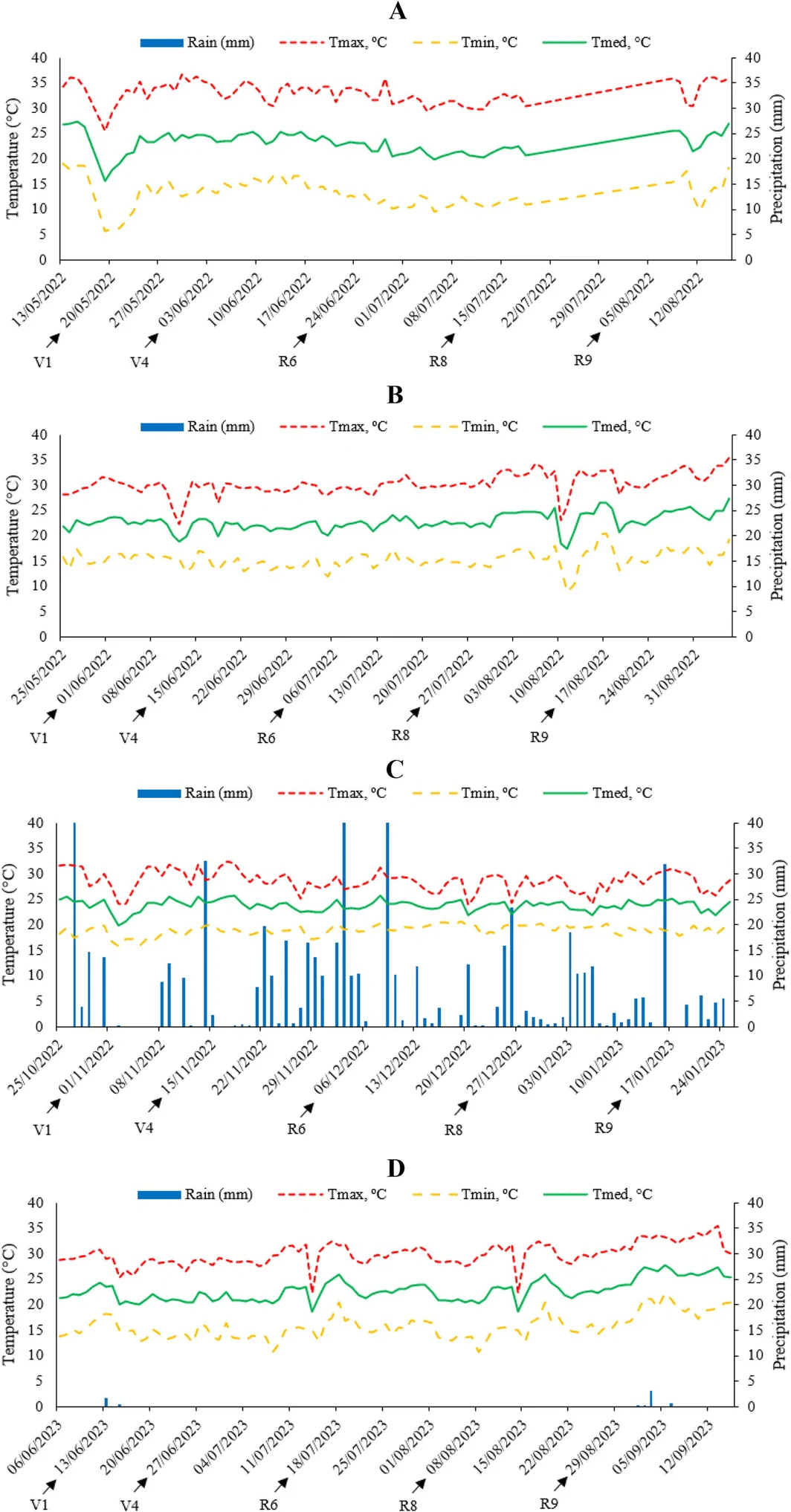
Precipitation and maximum, minimum and average temperatures recorded during the experiments, along with the respective phenological stages: R6, flowering; R8, pod filling; R9, ripening; V1, emergence; V4, third trifoliate leaf. Ceres‐GO, winter 2022 (A); Santo Antônio de Goiás‐GO, winter 2022 (B); Santo Antônio de Goiás‐GO, rainy season 2022/2023 (C); Santo Antônio de Goiás‐GO, winter 2023 (D).

Precipitation data, along with the maximum, minimum and average temperatures recorded during the experiments, are presented in Figure 1.

Prior to establishing the experiments, soil samples were collected from a depth of 0–20 cm to assess soil texture and fertility. Physical and chemical analyses were performed following the methodology proposed by Claessen (1997). The results of the chemical analysis are presented in Table 1. Textural analysis showed that the soils in Ceres and Santo Antônio contained 56% and 54% clay, 8% and 12% silt and 36% and 34% sand, respectively.

**TABLE 1:** Chemical attributes of the soil in the experimental areas in Santo Antônio de Goiás—GO (SAG) and Ceres—GO (CER).

Areas	pH	Ca	Mg	Al	H + Al	P	K	OM
	H_2_O	cmol_c_ dm^−3^				mg dm^−3^		g kg^−1^
SAG	5.1	2.4	1.1	0.0	3.0	14.2	77.1	25.4
CER	5.2	2.9	1.4	0.0	3.0	41.4	175.5	15.1

### 2.2 Design and Installation of Experiments

The experiments were conducted under field conditions using a randomized block design with 22 treatments and four replications. Each treatment consisted of a multifunctional combination of three plant growth‐promoting bacteria, in addition to control treatments. The bacterial strains used in the study are maintained in the Multifunctional Microorganisms Collection at Embrapa Rice and Beans and are described in Table 2, along with their main characteristics.

**TABLE 2:** Characteristics of the rhizobacterial strains used in this study.

Collection code	Bacterial strain	Species	Action mechanism	Geographical origin	References
922	PaJuG2R5	*Rhizobium* sp.	Biological N fixation	Minas Gerais—Brazil	Santos et al. (2023)
1154	ItiCaG1R4	*Rhizobium* sp.	Biological N fixation	São Paulo—Brazil	Santos et al. (2023)
1236	TilvG1R2	*Rhizobium* sp.	Biological N fixation	Paraná ‐ Brazil	Santos et al. (2023)
Commercial inoculant	SEMIA4077	*Rhizobium tropici*	Biological N fixation	Colombia	Martínez‐Romero et al. (1991)
1301	BRM063573	*Bacillus*	P solubilization	Bahia ‐ Brazil	Asobia (2023)
		*pumilus*			
1254	BRM067205	*Paenibacillus pabuli*	P solubilization	Paraná ‐ Brazil	Asobia (2023)
S22	BRM067207	*Bacillus subtilis*	P solubilization	Goiás ‐ Brazil	Braga et al. (2018)
Commercial inoculant	BRM034840 + BRM033112	*Bacillus subtilis + Bacillus megaterium*	P solubilization	Minas Gerais ‐ Brazil	(Oliveira et al. 2009)
1381	BRM063574	*Stenotrophomonas maltophilia*	IAA production	Paraná ‐ Brazil	Asobia (2023)
1341	BRM067206	*Bacillus pumilus*	IAA production	Bahia ‐ Brazil	Asobia (2023)
Commercial inoculant	Ab‐V5	*Azospirillum brasilense*	IAA production	Paraná ‐ Brazil	Hungria et al. (2018)

### 2.3 Seed Inoculation

Prior to sowing, the bean seeds were treated with liquid inoculants at a recommended rate of 300 mL ha^−1^. Inoculation was carried out based on the previously established bacterial consortia (Table 3). The bacterial suspensions were prepared at the Agricultural Microbiology Laboratory at Embrapa Rice and Beans, containing 1 × 10^8^ CFU mL^−1^ of each microorganism. The inoculation process was performed in the shade, and after the seeds had dried, they were stored in a cold chamber. Sowing was conducted the day after inoculation.

**TABLE 3:** Identification of treatments with the established combinations of bacterial strains, described by their respective strain codes.

Treatments	N‐fixing strains	P‐solubilizing strains	IAA‐producing strains
1	PaJuG2R5	BRM063573	BRM063574
2	PaJuG2R5	BRM063573	BRM067206
3	PaJuG2R5	BRM067205	BRM063574
4	PaJuG2R5	BRM067205	BRM067206
5	PaJuG2R5	BRM067207	BRM063574
6	PaJuG2R5	BRM067207	BRM067206
7	ItiCaG1R4	BRM063573	BRM063574
8	ItiCaG1R4	BRM063573	BRM067206
9	ItiCaG1R4	BRM067205	BRM063574
10	ItiCaG1R4	BRM067205	BRM067206
11	ItiCaG1R4	BRM067207	BRM063574
12	ItiCaG1R4	BRM067207	BRM067206
13	TilvG1R2	BRM063573	BRM063574
14	TilvG1R2	BRM063573	BRM067206
15	TilvG1R2	BRM067205	BRM063574
16	TilvG1R2	BRM067205	BRM067206
17	TilvG1R2	BRM067207	BRM063574
18	TilvG1R2	BRM067207	BRM067206
19	T0‐absolute control (no inoculation and no fertilization)		
20	T50‐reduced fertilization (45 kg ha^−1^ P; 15 kg ha^−1^ K; 6 kg ha^−1^ N)		
21	T100‐conventional fertilization (90 kg ha^−1^ P; 30 kg ha^−1^ K; 80 kg ha^−1^ N)		
22	TC‐commercial inoculants mix (SEMIA 4077 + BiomaPhos+Ab‐V5)		

### 2.4 Planting the Crop

Sowings were carried out during the third season of 2022 and 2023 (winter season), and the first season of 2022/2023 (rainy season), using the BRS FC402 bean cultivar. Planting was performed mechanically, distributing 15 seeds per meter with 0.45 m spacing between rows. Each plot consisted of 5 rows, 6 m in length, totalling 13.5 m^2^.

Fertilization was based on the results of the soil chemical analysis and followed technical recommendations for the crop. In the T100 plots (control treatment with conventional fertilization), 300 kg ha^−1^ of the 4–30–10 NPK formulation was applied at sowing, and 150 kg ha^−1^ of urea was applied as a topdressing 25 days after plant emergence. The remaining plots, except for T0 (absolute control), received 50% of the recommended NPK (150 kg ha^−1^) and no topdressing.

During the winter season, irrigation was applied through a centre pivot according to crop water requirements, totalling approximately 400 mm throughout the bean growth cycle.

Phytosanitary treatments were applied in accordance with crop recommendations at each developmental stage. Pre‐ and post‐emergent herbicides were used, and pests and diseases were controlled with crop‐specific insecticides and fungicides.

### 2.5 Evaluations and Statistical Analysis

At the R5 developmental stage (beginning of flowering), relative chlorophyll content was measured on three plants per plot using a Minolta SPAD‐502 m. Immediately afterward, three plants per plot were collected using a straight shovel to remove the root system to a depth of 25 cm and within a 25 cm radius, in order to evaluate the number of nodules, dry mass of nodules, shoot dry mass, and the length, surface area, diameter, volume and dry mass of the root system.

Roots were separated from the shoots, washed, and the nodules detached and counted. Roots were then photographed for morphological analysis using WinRHIZO software (Arsenault et al. 1995). Subsequently, nodules, shoots and roots were placed in paper bags and dried in a forced‐air oven at 65°C before being weighed on an analytical balance.

The dry shoot biomass was ground to determine total nitrogen and phosphorus contents, following the methodology described by Malavolta et al. (1989). Nutrient use efficiency was estimated by the ratio between dry biomass and total nutrient content, using the biological utilization coefficient (CUB) method described by De Barros et al. (1986).

At the R9 stage (physiological maturity), 10 plants were sampled per plot to evaluate yield components, including the pods and grains number. Yield was estimated by harvesting all plants from the central area of each plot, totalling 5.4 m^2^, and weighing the grain yield in kg ha^−1^, corrected to 13% moisture.

Data were subjected to multivariate analysis using R software (R Core Team 2023). Principal component analysis (PCA) was performed to describe correlations between treatments and response variables, and cluster analysis (dendrogram) was used to explore the similarity between treatments. The FactoMineR (Lê et al. 2008), factoextra (Kassambara and Mundt 2020), and cluster (Maechler 2022) packages were used.

Yield data were subjected to analysis of variance (ANOVA), and prior to mean comparison, model assumptions were verified through residual analysis, including assessment of normality using the Shapiro–Wilk test and homoscedasticity through inspection of residual plots. When significant differences were found, means were compared using the Scott‐Knott test at a 5% significance level.

## 3 Results

The growth variables and production components of common bean were influenced by the rhizobacteria consortia and the growing conditions. The specificities of the rhizobacterial combinations across locations and planting seasons highlighted the influence of soil and climate conditions on the outcomes.

The correlation coefficients between the variables varied across cultivation sites and crop seasons. The most relevant variables presented correlation values greater than 0.6, regardless of the sign, with correlations of the same sign indicating a positive relationship between the variables (Ferraudo 2014). It was observed that in the PCA1 of all seasons, the variables SDM, PUE and NUE were the most relevant. However, the production components (PN, GN and GY) varied among the principal components, showing greater relevance in the PCA1 of Ceres (winter 2022) and in the PCA2 of Santo Antônio (water 2022/2023) (Table 4).

**TABLE 4:** Correlation coefficients between the variables and the principal components PCA1 and PCA2 for the experiments conducted in two locations (Ceres—CE and Santo Antônio de Goiás—SAG) across three seasons (winter 2022, water 2022/2023 and winter 2023).

Variables	CE winter 22		SAG winter 22		SAG water 22/23		SAG winter 23	
	PCA1	PCA2	PCA1	PCA2	PCA1	PCA2	PCA1	PCA2
NN	0.277	−0.621	−0.208	−0.896	−0.372	0.266	−0.244	−0.443
NDM	0.385	−0.652	−0.148	−0.776	−0.366	0.100	−0.534	−0.313
RDM	−0.342	−0.335	−0.863	−0.150	−0.763	−0.309	−0.666	0.093
RL	−0.201	−0.547	−0.712	−0.036	0.672	−0.080	0.059	0.720
RSA	−0.338	−0.422	−0.759	0.390	0.631	−0.476	0.242	0.733
RV	−0.480	0.392	−0.434	0.400	−0.373	−0.474	−0.041	0.426
RD	−0.270	0.742	0.141	0.453	−0.325	−0.428	0.329	0.629
PN	0.642	−0.074	−0.124	0.608	0.057	−0.691	−0.044	−0.231
GN	0.692	−0.033	−0.226	0.610	0.103	−0.811	−0.419	−0.389
GY	0.625	−0.203	0.049	−0.279	−0.066	−0.719	0.062	−0.064
RCC	0.277	−0.297	0.047	−0.134	−0.255	−0.465	0.083	−0.348
SDM	−0.727	−0.474	−0.931	−0.139	−0.968	0.111	−0.860	0.422
TPCS	−0.275	0.429	−0.131	0.499	0.454	0.467	−0.385	−0.141
PUE	−0.839	−0.221	−0.884	0.092	−0.941	0.213	−0.896	0.377
TNCS	−0.054	0.174	−0.281	−0.186	0.463	0.329	−0.453	−0.364
NUE	−0.762	−0.423	−0.923	−0.159	−0.946	0.171	−0.910	0.309

Abbreviations: GN, grains number; GY, grain yield; NDM, nodule dry mass; NN, number of nodules; NUE, nitrogen use efficiency; PN, pods number; PUE, phosphorus use efficiency; RCC, relative chlorophyll content; RD, root diameter; RDM, root dry mass; RL, root length; RSA, root surface area; RV, root volume; SDM, shoot dry mass; TNCS, total nitrogen content shoot; TPCS, total phosphorus content shoot.

### 3.1 Ceres—Winter Season 2022

**FIGURE 2:**
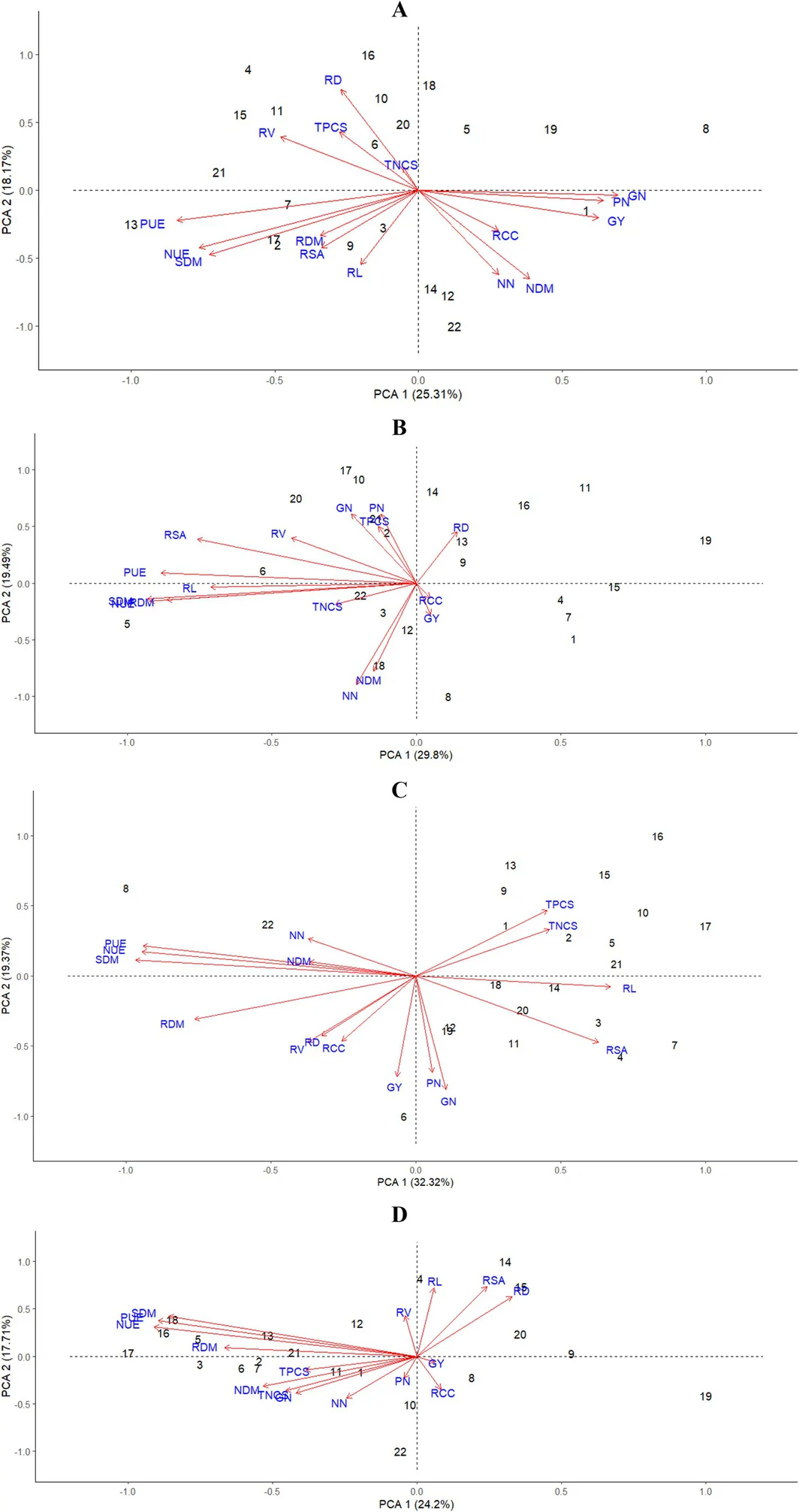
Principal component analysis (PCA) explaining the correlations between the variables and treatments (1—PaJuG2R5 + BRM063573 + BRM063574; 2—PaJuG2R5 + BRM063573 + BRM067206; 3—PaJuG2R5 + BRM067205 + BRM063574; 4—PaJuG2R5 + BRM067205 + BRM067206; 5—PaJuG2R5 + BRM067207 + BRM063574; 6—PaJuG2R5 + BRM067207 + BRM067206; 7—ItiCaG1R4 + BRM063573 + BRM063574; 8—ItiCaG1R4 + BRM063573 + BRM067206; 9—ItiCaG1R4 + BRM067205 + BRM063574; 10—ItiCaG1R4 + BRM067205 + BRM067206; 11—ItiCaG1R4 + BRM067207 + BRM063574; 12—ItiCaG1R4 + BRM067207 + BRM067206; 13—TilvG1R2 + BRM063573 + BRM063574; 14—TilvG1R2 + BRM063573 + BRM067206; 15—TilvG1R2 + BRM067205 + BRM063574; 16—TilvG1R2 + BRM067205 + BRM067206; 17—TilvG1R2 + BRM067207 + BRM063574; 18—TilvG1R2 + BRM067207 + BRM067206; 19—T0; 20—T50; 21—T100; 22—TC) for common beans grown in Ceres‐GO during the winter 2022 season (A), Santo Antônio de Goiás‐GO during the winter 2022 season (B), Santo Antônio de Goiás‐GO during the 2022/2023 water season (C), Santo Antônio de Goiás‐GO during the winter 2023 season (D).

The PCA conducted in Ceres‐GO (winter season 2022) resulted in two principal components with eigenvalues greater than 1, explaining 43.48% of the total data variation (Figure 2A). PCA1 accounted for 25.31% of this variation, with the variables pods number (PN), grains number (GN), grain yield (GY), shoot dry mass (SDM), phosphorus use efficiency (PUE) and nitrogen use efficiency (NUE) being the most relevant (Table 4).

It was observed that root dry mass (RDM), root length (RL) and root surface area (RSA) contributed to the variation in SDM, PUE and NUE. Root diameter (RD), root volume (RV), total nitrogen content shoot (TNCS) and total phosphorus content shoot (TPCS) exhibited a low positive correlation in PCA2, while number of nodules (NN), nodule dry mass (NDM) and relative chlorophyll content (RCC) were not correlated with these variables. Treatment 1 showed a strong correlation with the production components PN, GN and GY. Additionally, PCA2 indicated that these production components were also correlated with SDM, PUE, NUE, RDM, RL and RSA (Figure 2A).

**FIGURE 3:**
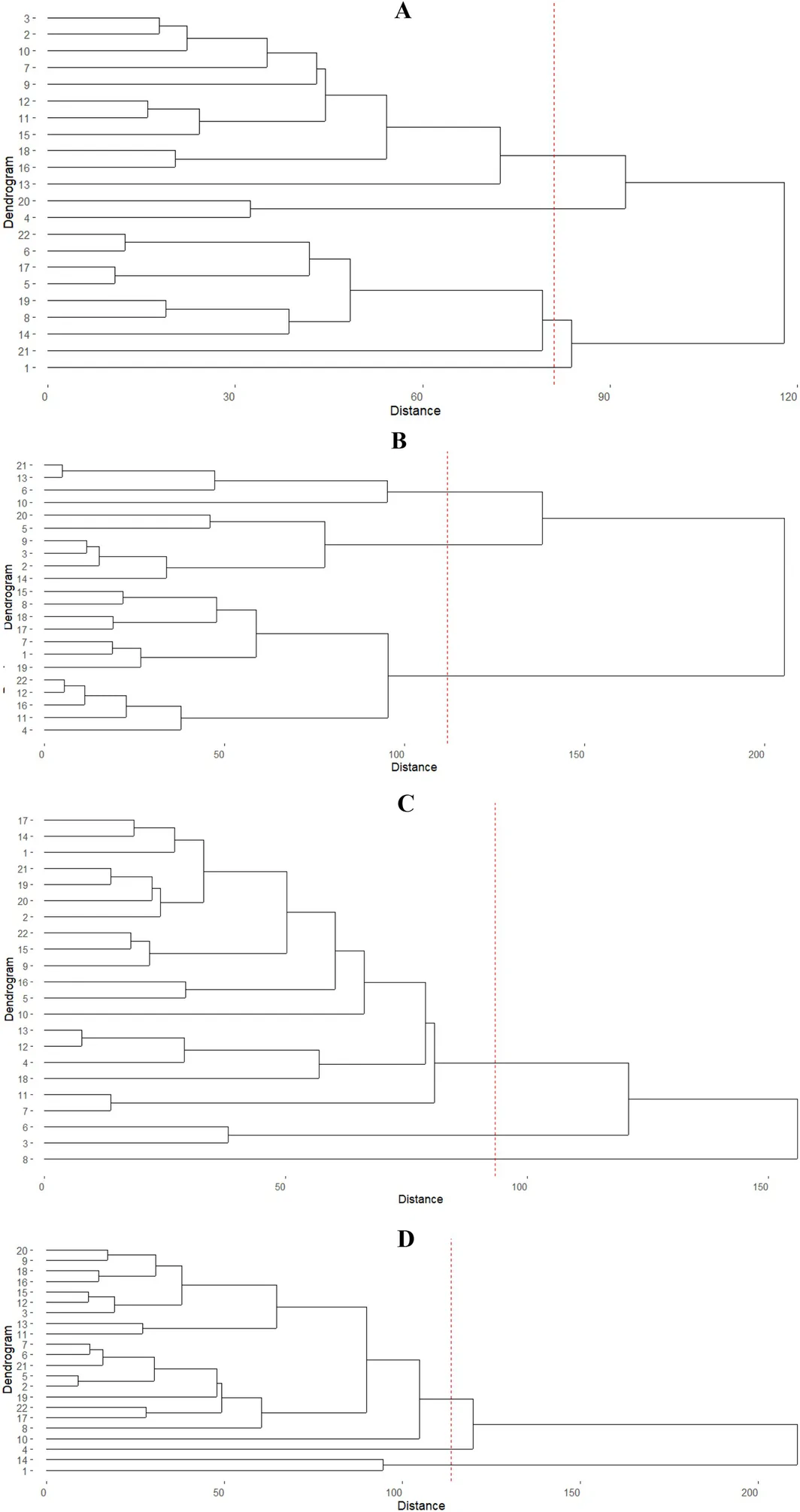
Cluster analysis (dendrogram) showing the similarity among common bean treatments based on agronomic, nutritional and productivity variables in Ceres‐GO during the winter 2022 season (A), Santo Antônio de Goiás‐GO during the winter 2022 season (B), Santo Antônio de Goiás‐GO during the 2022/2023 water season (C), Santo Antônio de Goiás‐GO during the winter 2023 season (D).

The 22 treatments were separated into four groups based on the studied variables. Treatment 1 stood out from the others, forming Group 1 individually. Group 2 included Treatments 5, 6, 8, 14, 17, 19, 21 and 22. Group 3 consisted of Treatments 4 and 20, while Group 4 comprised Treatments 2, 3, 7, 9, 10, 11, 12, 13, 15, 16 and 18 (Figure 3A).

### 3.2 Santo Antônio de Goiás—Winter Season 2022

In the Santo Antônio de Goiás experiment (winter crop 2022), the analysis resulted in two principal components with eigenvalues greater than 1, explaining 49.29% of the total data variation (Figure 2B). PCA1, which accounted for 29.8% of this variation, identified RDM, RL, RSA, SDM, PUE and NUE as the most relevant variables (Table 4).

Treatments 5 and 6 contributed directly to the strong correlations observed for RDM, RL, RSA, SDM, PUE and NUE. PCA1 also showed that these variables were positively correlated with NN, NDM, TNCS, RV, TPCS, PN and GY. Grain yield (GY) exhibited no significant coefficient and was negatively correlated with PN and GN. However, PCA2 indicated that NN, NDM, TNCS, RCC, RDM, SDM, NUE and RL contributed to grain yield (Figure 2B).

Based on the variables under study, the treatments were divided into three groups. Group 1 included Treatments 1, 4, 7, 8, 11, 12, 15, 16, 17, 18, 19 and 22, which exhibited similar characteristics. Group 2 consisted of Treatments 2, 3, 5, 9, 14 and 20. Group 3 comprised Treatments 6, 10, 13 and 21 (Figure 3B).

### 3.3 Santo Antônio de Goiás—Winter Season 2022/2023

The principal components PCA1 and PCA2 had eigenvalues greater than 1 and explained 51.69% of the variation in the data for the crop grown in Santo Antônio de Goiás (winter season 2022/2023) (Figure 2C). The variables RDM, RL, RSA, SDM, PUE and NUE were the most relevant in PCA1, which accounted for 32.3% of the variation (Table 4).

PCA1 showed that Treatments 3, 4 and 7 contributed to the strong positive correlation of the RL and RSA variables. Treatment 8 was more specific in its contribution to the SDM, PUE and NUE variables. Treatment 6 correlated with the production components (PN, GN and GY), and grain yield (GY) was influenced by the variables RCC, RD, RV, RDM, SDM, PUE, NUE, NN and NDM (Figure 2C).

The cluster analysis revealed the formation of three similarity groups. Treatment 8 alone formed Group 1, while Treatments 3 and 6 made up Group 2. Group 3 consisted of the remaining treatments (1, 2, 4, 5, 7, 9, 10, 11, 12, 13, 14, 15, 16, 17, 18, 19, 20, 21 and 22), which showed similarity based on the variables studied (Figure 3C).

### 3.4 Santo Antônio de Goiás—Winter Season 2023

In Santo Antônio de Goiás (winter season 2023), the PCA resulted in two main components (PCA1 and PCA2) with eigenvalues greater than 1, explaining 41.91% of the variation in the data (Figure 2D). The most relevant variables in PCA1, which accounted for 24.2% of the variation, were RDM, SDM, PUE and NUE (Table 4).

Treatments 4, 14 and 15 contributed to the strong positive correlation of the RL, RSA and RV variables. In PCA1, these variables were correlated with grain yield (GY), although with a low correlation coefficient. Treatments 1 and 11 specifically contributed to the NN, NDM, TCPS, TNCS, PN and GN variables, which also correlated with RDM, RD and more strongly with SDM, PUE and NUE. These last three variables were particularly influenced by Treatments 16, 17 and 18 (Figure 2D).

Based on the variables studied, the treatments were divided into three groups. Group 1 consisted of Treatments 1 and 14, while Group 2 was made up solely of Treatment 4. The remaining treatments (2, 3, 5, 6, 7, 8, 9, 10, 11, 12, 13, 15, 16, 17, 18, 19, 20, 21 and 22) were similar and comprised Group 3 (Figure 3D).

### 3.5 Grain Yield

Grain yield of common beans showed significant differences among treatments and varied according to the locations and growing seasons. In Ceres, during the winter 2022 season, Treatments 1, 8 and 14 resulted in higher yields compared to the other treatments, although they did not differ statistically from one another. In Santo Antônio de Goiás, in the winter 2022 season, 13 treatments (1, 2, 3, 5, 7, 8, 9, 14, 15, 17, 18, 19 and 20) exhibited statistically similar yields, achieving higher values than the other treatments. During the 2022/2023 rainy season, grain yield was higher in Treatments 3 and 6. In the winter 2023 season, Treatments 1 and 14 achieved the highest yields and were statistically superior to the others, which did not differ among themselves (Table 5).

**TABLE 5:** Mean grain yield values (kg ha^−1^) of common beans grown in Ceres‐CE and Santo Antônio de Goiás‐SAG during the 2022 winter, 2022/2023 rainy and 2023 winter seasons.

Treat.	CE winter 22	SAG winter 22	SAG water 22/23	SAG winter 23
1	3285.7a	1571.9a	1493.0c	3464.3a
2	2687.6c	1598.9a	1515.8c	2866.9b
3	2623.0c	1607.3a	1932.8a	2545.6b
4	2614.7c	1430.0b	1715.3b	2505.6b
5	2938.0b	1662.6a	1362.4d	2873.9b
6	2887.7b	1300.0b	2075.1a	2803.1b
7	2768.5c	1637.5a	1707.4b	2803.6b
8	3083.6a	1598.7a	1334.4d	2929.9b
9	2596.8c	1649.9a	1487.5c	2653.7b
10	2623.8c	1278.4b	1362.7d	2586.6
11	2505.9c	1372.3b	1653.5b	2319.5b
12	2545.2c	1434.4b	1716.0b	2519.8b
13	2374.5c	1261.5b	1724.1b	2397.8b
14	3127.1a	1707.0a	1424.9d	3443.7a
15	2497.3c	1515.8a	1512.0c	2473.6b
16	2620.6c	1407.0b	1262.6d	2666.2b
17	2966.9b	1698.0a	1481.3c	2893.7b
18	2698.6c	1699.4a	1823.6b	2614.2b
19	3020.6b	1670.6a	1584.5c	2731.9b
20	2505.2c	1515.5a	1611.1c	2634.9b
21	2931.0b	1257.0b	1553.1c	2787.8b
22	2905.4b	1424.8b	1562.5c	2977.7b
CV%	6.1	10.6	8.6	11.0

*Note:* Means followed by the same lowercase letter within a column do not differ statistically according to the Scott‐Knott test at a 5% probability level. Treatments: 1—PaJuG2R5 + BRM063573 + BRM063574; 2—PaJuG2R5 + BRM063573 + BRM067206; 3—PaJuG2R5 + BRM067205 + BRM063574; 4—PaJuG2R5 + BRM067205 + BRM067206; 5—PaJuG2R5 + BRM067207 + BRM063574; 6—PaJuG2R5 + BRM067207 + BRM067206; 7—ItiCaG1R4 + BRM063573 + BRM063574; 8—ItiCaG1R4 + BRM063573 + BRM067206; 9—ItiCaG1R4 + BRM067205 + BRM063574; 10—ItiCaG1R4 + BRM067205 + BRM067206; 11—ItiCaG1R4 + BRM067207 + BRM063574; 12—ItiCaG1R4 + BRM067207 + BRM067206; 13—TilvG1R2 + BRM063573 + BRM063574; 14—TilvG1R2 + BRM063573 + BRM067206; 15—TilvG1R2 + BRM067205 + BRM063574; 16—TilvG1R2 + BRM067205 + BRM067206; 17—TilvG1R2 + BRM067207 + BRM063574; 18—TilvG1R2 + BRM067207 + BRM067206; 19—T0; 20—T50; 21—T100; 22—TC.

## 4 Discussion

The results demonstrated that the combined use of rhizobacterial consortia was effective in promoting the growth and grain yield of common beans, enabling a reduction in mineral fertilization. The superior performance of plant growth‐promoting rhizobacteria (PGPR) compared to mineral fertilization highlights the potential benefits of these microbial consortia in common bean production systems. However, it was observed that the effectiveness of the consortia varied according to location and growing season. This underscores the specificity of microbial strains and their interaction with soil and climatic conditions, particularly due to fluctuations in environmental factors such as temperature, humidity and solar radiation, which can affect microbial activity.

In Ceres, during the 2022 winter season, the best performance was observed with the consortium *Rhizobium* sp. (PaJuG2R5) + *Bacillus pumilus* (BRM063573) + *Stenotrophomonas maltophilia* (BRM063574) in Treatment 1. In Santo Antônio de Goiás, in the same season, the consortia *Rhizobium* sp. (PaJuG2R5) + *Bacillus subtilis* (BRM067207) + *Stenotrophomonas maltophilia* (BRM063574) in Treatment 5, and *Rhizobium* sp. (PaJuG2R5) + *Bacillus subtilis* (BRM067207) + *Bacillus pumilus* (BRM067206) in Treatment 6, had a strong influence on key variables (RL, RSA, SDM, PUE and NUE), which were correlated with PN and GN. However, the favourable performance of these variables did not translate into higher grain yield, possibly due to the occurrence of angular leaf spot, caused by *Pseudocercospora griseola*, during the grain maturation stage.

In the 2022/2023 rainy season, the consortium *Rhizobium* sp. (PaJuG2R5) + *Bacillus subtilis* (BRM067207) + *Bacillus pumilus* (BRM067206) in Treatment 6 showed influence over PN, GN and GY, which were correlated with root system variables. In this season, the consortia *Rhizobium* sp. (PaJuG2R5) + *Paenibacillus pabuli* (BRM067205) + *Stenotrophomonas maltophilia* (BRM063574), in Treatment 3, and *Rhizobium* sp. (ItiCaG1R4) + *Bacillus pumilus* (BRM063573) + *Bacillus pumilus* (BRM067206) in Treatment 8 influenced both root and shoot development variables.

In the 2023 winter season, Treatments 1 (*Rhizobium* sp. (PaJuG2R5) + *B. pumilus* + *S. maltophilia* ) and 14 (*Rhizobium* sp. (TilvG1R2) + *B. pumilus* + *B. pumilus* ) provided the most significant contributions to plant performance.

Variables related to root development showed high coefficients, highlighting the effectiveness of rhizobacteria producing phytohormones, particularly indole‐3‐acetic acid (IAA), which directly influences root architecture. Several bacterial genera can synthesize phytohormones that promote cell elongation and division, stem and root growth, and the formation of lateral and adventitious roots. These effects improve water and nutrient uptake, ultimately enhancing overall plant growth (Etesami and Maheshwari 2018).

To achieve these beneficial effects via IAA production, both efficient transport of the hormone to target cells and proper perception by cellular membranes are required. During long photoperiods, endogenous auxin synthesis naturally increases, potentially enhancing the effects of exogenous auxins produced by rhizobacteria. Conversely, during winter cultivation, characterized by shorter days, endogenous auxin production is reduced, which may limit the efficacy of bacterial inoculants (Shaffique et al. 2023).

Treatments containing *Stenotrophomonas maltophilia* and *Bacillus pumilus* were the most effective in enhancing root development variables across cultivation sites, underscoring their potential in symbiotic interactions and seasonal adaptation via IAA production. Cruz et al. (2024) also reported the effectiveness of similar bacterial consortia in promoting root and shoot development in maize seedlings, while Shaffique et al. (2023) highlighted the positive impact of *B. pumilus* on soybean growth.

In Ceres (winter 2022) and in Santo Antônio de Goiás (rainy season 2022/2023), root area and length were positively correlated with nitrogen (N) and total phosphorus (P) content. In Ceres, root volume and diameter also showed positive correlations with nutrient content. These results reflect the capacity of improved root architecture to enhance soil exploration, facilitating deeper penetration and increased soil volume exploitation, which leads to improved nutrient uptake (van der Bom et al. 2020).

Additionally, the action mechanisms of phosphate‐solubilizing rhizobacteria proved effective in increasing P uptake by the plants. Considering that the recommended phosphorus content for common beans ranges from 2 to 3 g kg^−1^ (Meneghetti 2018), the results were satisfactory, with mean values exceeding 3 g kg^−1^ in all cropping seasons.

Despite the high total phosphorus stocks in the experimental soils, less than 0.1% is considered readily available for plant absorption (Filiz et al. 2022). Moreover, efficient phosphorus uptake and transport require favourable environmental conditions, such as soil temperatures around 30°C and adequate moisture (Rengel et al. 2022). Thus, the consortia containing *Bacillus* strains were effective in hydrolysing organic and inorganic phosphorus compounds, improving P availability under the soil and climatic conditions encountered in this study.

Across all results, the high relevance of nitrogen and phosphorus use efficiency and shoot biomass was evident. Both P and N are essential macronutrients involved in nearly all physiological and biochemical processes of plant development, and they constitute a significant proportion of plant dry matter (Kalayu 2019). The bacterial consortia employed in this study significantly enhanced nutrient use efficiency and biomass accumulation. However, improvements in the uptake and utilization of P and N did not translate proportionally into increased grain yield. A similar pattern was reported by Galindo et al. (2024) in maize, who associated the limited conversion of absorbed P and N into grains with the potential limitation of other essential nutrients or adverse environmental conditions.

Micronutrients such as Zn, Fe, Mn and Mo are required as cofactors in enzymatic pathways associated with photosynthesis, nitrogen metabolism, and reproductive development, and their limitation can constrain grain filling even when N and P uptake is enhanced. Consequently, improvements in macronutrient acquisition do not necessarily translate into proportional yield increases when micronutrient supply is suboptimal. In addition, PGPR‐driven stimulation of vegetative growth may induce carbon allocation trade‐offs, whereby increased biomass accumulation and root activity occur at the expense of assimilate partitioning toward reproductive organs. Furthermore, biotic pressures and interactions within the rhizosphere may further influence plant resource allocation, diverting energy toward maintenance and defence rather than grain production (Vaghela et al. 2025).

Regarding plant nodulation, the number of nodules observed in Ceres remained below 15 in all treatments. This falls short of the benchmark for adequate nodulation, typically considered between 15 and 30 nodules or 100–200 mg of nodule dry mass per plant (Horácio et al. 2020). Several factors may have contributed to this outcome. Climatic and nutritional conditions, as well as microbial competitiveness in the rhizosphere, may have hindered the effective colonization of roots by *Rhizobium* strains. The high soil fertility in Ceres could have suppressed nodulation, as elevated soil nitrogen levels reduce the plant's reliance on biological nitrogen fixation, thereby diminishing the rhizobacteria's incentive to establish nodules (Messias et al. 2023). Moreover, maximum temperatures in Ceres exceeded 35°C (Figure 1A), a condition potentially detrimental to the survival and activity of *Rhizobium* strains, especially given that these strains were originally isolated from regions with milder climates (Table 2). Environmental variables such as pH, temperature, humidity, salinity, nitrogen availability, elevation, and latitude have been reported to significantly affect the symbiotic efficiency of rhizobia and the responsiveness of common beans to inoculation (Gunnabo et al. 2019).

In the other cropping seasons in Santo Antônio de Goiás, plants exhibited adequate nodulation but low nodule dry mass. Nevertheless, the symbiotic efficiency of rhizobia was evidenced by the positive correlation between nodulation and both chlorophyll content and grain yield. The chlorophyll index is a reliable indicator of nitrogen deficiency, with SPAD values above 45 considered optimal (Matoso et al. 2021). PCA revealed that Treatment 22, which included the bacterial strain *Rhizobium tropici* SEMIA4077, had the most substantial influence on the NN and NDM variables. This strain, originally isolated from Colombia, has shown strong nitrogen‐fixing potential under Brazilian field conditions and is widely used in commercial inoculants for common beans (Horácio et al. 2020).

In Ceres, during the 2022 winter season, common bean yield exceeded 3000 kg ha^−1^ in treatments with *Rhizobium* sp. (PaJuG2R5) + *Bacillus pumilus* (BRM063573) + *Stenotrophomonas maltophilia* (BRM063574) − Treatment 1; *Rhizobium* sp. (ItiCaG1R4) + *Bacillus pumilus* (BRM063573) + *Bacillus pumilus* (BRM067206) − Treatment 8; and *Rhizobium* sp. (TilvG1R2) + *Bacillus pumilus* (BRM063573) + *Bacillus pumilus* (BRM067206) −Treatment 14. Treatment 1 alone outperformed conventional fertilization by over 10%, translating to nearly six additional 60 kg bags per hectare. Similar results were reported by Messias et al. (2023), who obtained yields above 3000 kg ha^−1^ using co‐inoculation with *Rhizobium tropici* and *Azospirillum brasilense* ; however, their yield increase over mineral fertilization was limited to just 1%.

In Santo Antônio de Goiás, during both the 2022 winter and 2022/2023 water seasons, high yields could not be achieved due to the occurrence of angular leaf spot caused by the fungus *Pseudocercospora griseola*. This disease is among the most severe for common beans and can reduce yield by up to 80% (Couto et al. 2008). Nevertheless, yields in both experiments remained above the national average of 1138 kg ha^−1^ (Conab 2024).

The association of plant growth‐promoting rhizobacteria may also contribute to disease suppression through antagonistic interactions with phytopathogens, thereby reducing disease severity (Li et al. 2020). In a similar context, Silva et al. (2023) reported low yields during the water season in co‐inoculated common bean trials. Environmental conditions, particularly temperature and humidity, can strongly affect pathogen incidence and crop productivity, and in 2023, the El Niño phenomenon significantly disrupted grain production due to climatic anomalies.

During the 2022 winter season, the consortium *Rhizobium* sp. (PaJuG2R5) + *Bacillus subtilis* (BRM067207) + *Stenotrophomonas maltophilia* (BRM063574) − Treatment 5, surpassed the yield of mineral fertilization by 32%. The consortium *Rhizobium* sp. (PaJuG2R5) + *Bacillus subtilis* (BRM067207) + *Bacillus pumilus* (BRM067206) − Treatment 6, matched conventional fertilization. Meanwhile, *Rhizobium* sp. (TilvG1R2) + *Bacillus pumilus* (BRM063573) + *Bacillus pumilus* (BRM067206) − Treatment 14, outperformed fertilization by 35%, resulting in a 450 kg ha^−1^ production increase.

In the 2022/2023 water season, the consortia *Rhizobium* sp. (PaJuG2R5) + *Paenibacillus pabuli* (BRM067205) + *Stenotrophomonas maltophilia* (BRM063574) − Treatment 3 and *Rhizobium* sp. (PaJuG2R5) + *Bacillus subtilis* (BRM067207) + *Bacillus pumilus* (BRM067206) − Treatment 6 yielded, on average, 30% more than mineral fertilization, representing productivity gains of up to 500 kg ha^−1^.

In the 2023 winter season, grain yields approached 3500 kg ha^−1^ with the bacterial consortia *Rhizobium* sp. (PaJuG2R5) + *Bacillus pumilus* (BRM063573) + *Stenotrophomonas maltophilia* (BRM063574) − Treatment 1 and *Rhizobium* sp. (TilvG1R2) + *Bacillus pumilus* (BRM063573) + *Bacillus pumilus* (BRM067206) − Treatment 14. These consortia outperformed both the commercial inoculant and conventional fertilization treatments, yielding on average eight more 60‐kg bags than the former and eleven more than the latter, representing productivity increases of 16% and 23%, respectively.

Messias et al. (2024) conducted an economic analysis of co‐inoculation in common beans and reported the highest production costs associated with nitrogen fertilization. Co‐inoculation, on the other hand, provided an 11% higher cost–benefit ratio than mineral fertilization, indicating that this strategy not only enhances yield but also contributes to greater profitability and reduced environmental impacts in common bean production.

Despite the specificity of rhizobacteria to cultivation site and season, certain strains consistently demonstrated greater efficacy and adaptability under the experimental conditions. In particular, *Rhizobium* sp. (PaJuG2R5), *Bacillus* spp. (BRM067207, BRM063573 and BRM067206), and *Stenotrophomonas maltophilia* (BRM063574) exhibited mechanisms of action that effectively supported plant growth and development. Similar findings were reported by Rezende (2024) in soybean, where co‐inoculation with *Bacillus* strains BRM063573 and BRM067206 led to positive agronomic responses. In a previous study, promising results were also observed in common bean grain production using combinations that included *Bacillus pumilus* (BRM063573) and *Stenotrophomonas maltophilia* (BRM063574) (Rezende et al. 2021).

In a broader context, the use of multifunctional crop‐livestock‐forestry systems aligns with global efforts aimed at reducing dependence on mineral fertilizers through sustainable intensification and improved nutrient use efficiency. These approaches are increasingly recognized as key components of environmentally responsible agricultural systems, especially in regions where fertilizer costs and environmental impacts limit productivity gains.

Beyond its agronomic implications, this study advances current knowledge of rhizosphere microbiology by demonstrating that multifunctional bacterial consortia can maintain complementary ecological functions under contrasting field environments. The differential performance of the consortia across locations and growing seasons highlights that microbial functionality is strongly context‐dependent, reflecting interactions among bacterial strains, plant hosts, soil properties and climatic conditions. These findings reinforce the concept that successful inoculant design should consider ecological compatibility among microorganisms and environmental adaptation rather than simply combining strains with desirable individual traits. Consequently, this work contributes to a better understanding of microbial interactions governing plant‐microbiome functioning under field conditions.

## 5 Conclusion

Consortia of multifunctional rhizobacteria have proven effective in enhancing the performance of common bean crops under the given growing conditions. The strains PaJuG2R5 (*Rhizobium* sp.), BRM067207 ( *Bacillus subtilis* ), BRM063573 ( *Bacillus pumilus* ), BRM067206 ( *Bacillus pumilus* ), and BRM063574 ( *Stenotrophomonas maltophilia* ) demonstrated greater efficiency and adaptability to the study conditions.

For winter season (third season), it is recommended to cultivate common beans using the consortia *Rhizobium* sp. (PaJuG2R5) + *Bacillus pumilus* (BRM063573) + *Stenotrophomonas maltophilia* (BRM063574) or *Rhizobium* sp. (TilvG1R2) + *Bacillus pumilus* (BRM063573) + *Bacillus pumilus* (BRM067206). During the rainy season (first season), the recommended consortium is *Rhizobium* sp. (PaJuG2R5) + *Bacillus subtilis* (BRM067207) + *Bacillus pumilus* (BRM067206).

Therefore, under these soil and climatic conditions, using consortia containing these rhizobacteria represents an effective strategy to support the growth, development, and grain yield of common bean crops while reducing the need for mineral fertilization. In addition to agronomic benefits, this approach has the potential to lower production costs and reduce environmental impacts, contributing to sustainable production systems, especially for family farming.

## Author Contributions

**Ana Paula Santos Oliveira:** conceptualization, data curation, formal analysis, investigation, methodology, visualization, writing – original draft, writing – review and editing. **Cássia Cristina Rezende Mirza:** conceptualization, formal analysis, methodology, visualization, writing – review and editing. **Cleiton Mateus Sousa:** formal analysis, methodology, visualization, writing – review and editing. **Enderson Petrônio de Brito Ferreira:** funding acquisition, formal analysis, investigation, supervision, validation, visualization, writing – review and editing.

## Funding

This work was supported by Empresa Brasileira de Pesquisa Agropecuária (20.20.00.061.00.00), INCT‐Plant Growth Promoting Microorganisms for Agricultural Sustainability and Environmental Responsibility (CNPq 465133/2014‐4, Fundação Araucária‐STI 043/2019, CAPES) and Conselho Nacional de Desenvolvimento Científico e Tecnológico (313827/2020‐6).

## Conflicts of Interest

The authors declare no conflicts of interest.

## Data Availability

The data that support the findings of this study are available on request from the corresponding author.
